# Working memory- and anxiety-related behavioral effects of repeated nicotine as a stressor: the role of cannabinoid receptors

**DOI:** 10.1186/1471-2202-14-20

**Published:** 2013-02-09

**Authors:** Tamaki Hayase

**Affiliations:** 1 Department of Legal Medicine, Kyoto University, Yoshidakonoe-cho, Sakyo-ku, Kyoto 606-8501, Japan

**Keywords:** Nicotine, Immobilization stress, Working memory, Anxiety, Cannabinoid, AM 251, CP 55,940, virodhamine

## Abstract

**Background:**

Like emotional symptoms such as anxiety, modulations in working memory are among the frequently-reported but controversial psychiatric symptoms associated with nicotine (NC) administration. In the present study, repeated NC-induced modulations in working memory, along with concurrently-observed anxiety-related behavioral alterations, were investigated in mice, and compared with the effects of a typical cognition-impairing stressor, immobilization stress (IM). Furthermore, considering the structural and functional contributions of brain cannabinoid (CB) receptors in NC-induced psychiatric symptoms including emotional symptoms, the interactive effects of brain CB receptor ligands (CB ligands) and NC and/or IM on the working memory- and anxiety-related behaviors were examined.

**Results:**

Statistically significant working memory impairment-like behavioral alterations in the Y-maze test and anxiety-like behavioral alterations in the elevated plus-maze (EPM) test were observed in the groups of mice treated with 0.8 mg/kg NC (subcutaneous (s.c.) 0.8 mg/kg treatment, 4 days) and/or IM (10 min treatment, 4 days). In the group of mice treated with NC plus IM (NC-IM group), an enhancement of the behavioral alterations was observed. Among the CB type 1 (CB1) antagonist AM 251 (AM), the non-selective CB agonist CP 55,940 (CP), and the CB1 partial agonist/antagonist virodhamine (VD), significant recovering effects were provided by AM (0.2-2.5 mg/kg) and VD (5 mg/kg) against the working memory impairment-like behaviors, whereas significant anxiolytic-like effects (recoveries from both attenuated percentage of entries into open arms and attenuated percentage of time spent on open arms) were provided by VD (1–10 mg/kg) and CP (2 mg/kg) against the anxiety-like behaviors.

**Conclusions:**

Although working memory impairment- and anxiety-like behavioral alterations were commonly induced in the NC, IM, and NC-IM groups and the therapeutic involvement of CB receptors was shown, there were discrepancies in the types of effective CB ligands between the working memory- and anxiety-related behaviors. The differential involvements of CB receptor subtypes and indirectly activated neurotransmitter systems may contribute to these discrepancies.

## Background

Nicotine (NC) is the substance which sustains the addictive use of tobacco, and tobacco results in numerous harmful health effects and continues to be the leading cause of preventable death [1,2]. It has been reported that addicted tobacco users suffer from NC-induced cognitive impairments in some conditions of smoking, as well as modulated moods such as anxiety- and depression-related symptoms [3-5]. Cognitive impairments including deficits in working memory, a process for maintaining temporary active information [6], have been regarded as being among the representative symptoms of NC withdrawal observed in NC-dependent human and rodent models [3,7,8]. Furthermore, the direct neurotoxic effects of NC have also been reported depending on the treatment conditions such as dose, period and paradigm, and this neurotoxicity has been suggested to induce memory impairments, particularly at earlier periods in development [9-12]. However, in some clinical and experimental animal studies, cognitive improvements or absence of any effects have been demonstrated [13-17]. Negative, positive or no effects of NC have also been reported against the anxiety-related behaviors [18-20].

Working memory impairments have been reported for various stressors such as restraint stress (immobilization stress) in both humans and rodent models [21-23]. Like certain NC treatments, such stressors also induce and exacerbate the anxiety-like behavioral responses in rodent models [24,25]. Furthermore, it has been suggested that brain regions such as the medial prefrontal cortex, for which NC-induced modulations have been demonstrated [26,27], are concurrently involved in the development of stress-induced working memory impairments and anxiety [28-31]. However, there are only a few studies investigating the characteristic effects of NC as a stressor, particularly those on cognitive function [32,33].

A considerable number of studies have implicated the relationship between NC and stress. For example, in some rodent models, repeated or acute stress has been shown to aggravate the behavioral and neuronal effects of NC [34-36]. Recent human studies have shown some directly-exacerbated mood symptoms induced by stress in smokers [37,38]. However, against the behavioral and neuronal impairments caused by stress, antagonistic effects of subsequently administered NC have been shown in some rodent models [39-41]. With respect to cognitive function, NC has also been reported to block stress-induced impairments in several experimental conditions in rodents [41,42]. Nevertheless, the above-mentioned neurotoxic effects of NC which could lead to cognitive dysfunction [10-12] may be correlated with the possibility that NC and stress augment each other’s unfavorable effects on cognitive function.

In previous studies, a strong involvement of brain cannabinoid (CB) receptors, typically CB type 1 (CB1) receptors, was reported in the representative emotion-related behaviors (anxiety- and depression-like behaviors) induced by NC [18,43,44] and stress [45] in rodents. This is consistent with the prominent behavioral alterations induced by NC in CB1 knockout mice [46], and the overlapping distribution of CB1 receptors and nicotinic acetylcholine receptors (nAChRs) in some brain regions which supports functional interactions between these receptors [47,48]. Furthermore, recent reviews suggest that CB1 receptors contribute to deficits in memory including working memory by demonstrating that CB1 agonists impair memory formation and CB1 antagonists reverse these impairments [49,50]. However, there have been a limited number of studies on the direct contribution of brain CB receptors to the memory-related effects of NC [51,52]. The participation of CB1 receptors has also been reported in anxiety processes, but the roles of CB1 agonist are contradictory in that both anxiolytic-like and anxiogenic-like effects have been induced depending on the treatment conditions [53,54]. Against the NC-induced anxiety-related behaviors, inconsistent and contradictory effects of CB1 agonists and other CB ligands have also been demonstrated [18,43].

In the present study, using behavioral tests in mice (Y-maze and elevated plus-maze (EPM) test), the working memory- and anxiety-related behavioral alterations caused by NC were assessed and compared with those caused by immobilization stress (IM), a typical stressor. The interactions between the NC- and IM-induced behavioral effects were also examined. Furthermore, considering the possible involvement of brain CB receptors in these NC- and/or IM-induced memory- and anxiety-related behavioral alterations, the effects of selected CB ligands (the CB1 antagonist AM 251, the non-selective CB agonist CP 55,940, and the CB1 partial agonist/antagonist virodhamine) were evaluated against these behavioral alterations, as described in previous studies [43,51,52].

## Methods

### Animals

Based on previous studies on NC and stressor treatments [43,55], male ICR mice (80 ± 10 days old) (Shizuoka Laboratory Animal Center, Hamamatsu, Japan) were housed in a forced-air facility, which was maintained at 23°C and 50% relative humidity, with a 12 h/12 h light/dark cycle. The mice were kept separately in single transparent cages measuring 23.5 × 16.5 × 12 cm, and were allowed water and rodent chow ad libitum. The experiments described in this report were conducted in accordance with the “Guidelines for Animal Experiments” of the institution (updated in 2007) [56], which are based on the National Institutes of Health Guide for the Care and Use of Laboratory Animals, and any pain experienced by the mice was minimized. These guidelines were approved by the institutional ethics committee for animal experiments [56]. All of the observations and evaluations were performed by a trained observer who was blinded to the treatment conditions and was not informed of the treatment conditions in advance. Each experimental group contained 10 mice.

### Drug and stressor treatments

The protocols for the NC and stressor treatments were determined based on preliminary experiments and previous studies [43,55,57]. With respect to NC, repeated subcutaneous (s.c.) doses of NC which caused the emotional behaviors (anxiety- and depression-like behaviors) effectively in mice [43] were selected: single s.c. doses of 0.3 or 0.8 mg/kg were administered daily for 4 days. NC (Nacalai Tesque, Inc., Kyoto, Japan) was supplied in free-base form at 95% purity, and was freshly dissolved in saline to a volume of 5 ml/kg immediately before each administration. With respect to the stressor, treatments using IM, which have also been demonstrated to cause these emotional behaviors in rodents [44,58], were used. In the present experiments, repeated IM treatments in which the effects were almost equivalent to the peak effects of the NC treatments in preliminary experiments were selected: 10 min of IM, which was induced by placing the mouse in a narrow space (diameter about 12 cm) in a vinyl bag with some breathing holes, was performed once per day for 4 days. Furthermore, to investigate the interactions between NC and IM, the behavioral alterations were examined in the NC plus IM group (NC-IM group) which received the above s.c. dose of NC 10 min before the IM treatment once per day for 4 days, according to a previous study [59].

The CB ligands AM 251 (N-(piperidin-1-yl)-5-(4-iodophenyl)-1-(2,4-dichlorophenyl)-4-methyl-1H-pyrazole-3-carboxamide) (AM), CP 55,940 ((−)-cis-3-[2-hydroxy-4-(1,1-dimethylheptyl)phenyl]-trans-4-(3-hydroxypropyl)cyclohexanol) (CP), and virodhamine (O-(2-aminoethyl)-5Z,8Z,11Z,14Z-eicosatetraenoate) (VD) were purchased from Tocris Cookson Inc. (Ellisville, Missouri, USA), and the doses were selected based on previous studies and preliminary experiments [43,51,52]. For each drug, the data were collected and shown for those intraperitoneal (i.p.) doses which induced no toxic behavioral alterations by themselves at the prescribed time point: 0.2, 1 and 2.5 mg/kg for AM, 0.5, 2 and 5 mg/kg for CP, and 1, 5 and 10 mg/kg for VD. The CB ligands were dissolved and diluted using a mixed solution of dimethylsulphoxide (DMSO) plus distilled water, and were administered in a volume of 5 ml/kg, 60 min before each NC, IM or NC-IM treatment, considering the previously examined time course of the effects of CB ligands against the NC- and/or IM-induced working memory- and anxiety-related behaviors [43,58]. Since VD was provided in an ethanol solution (Tocris Cookson Inc.), the ethanol was evaporated immediately before use under nitrogen gas, and the residue was re-suspended in the same mixed DMSO/distilled water solution. In the NC- and IM-only groups, a mixed vehicle solution of DMSO and distilled water at the same ratio as the CB ligand solutions was injected instead of the CB ligands. In the CB ligand-only groups, the same volume of saline vehicle was injected instead of the NC or IM treatment. In the control group without any drug or stressor treatment (control group), the mixed vehicle solution of DMSO and distilled water was injected instead of the CB ligands, and then the same volume of saline vehicle was injected instead of the NC or IM treatment. The drug and stressor treatments and each experimental session were performed between 15 and 19 h of the light cycle.

### Y-maze test

Based on previous studies [28,60,61], alterations in working memory-related behaviors were examined in the Y-maze test using a cardboard apparatus that consisted of three enclosed arms 30 × 5 × 15 cm (length, width, and height) which converged on an equilateral triangular center platform (5 × 5 × 5 cm). After the number of spontaneous alteration performance (SAP), which was defined as the number of successive triplet entry performances into each of the three arms without any repeated entries [28,60,61], and the total number of entries into arms were evaluated (8 min test periods), the rate of spontaneous alteration performance (SAP rate) (%) was calculated as a parameter for the working memory-related behaviors. The total number of entries into arms was assessed as a parameter representing locomotor activity [60,61]. Considering the previous data [58], the evaluations of these parameters were performed at the 2 h time point after the last NC, IM or NC-IM treatment. At the beginning of each experimental session, each mouse was placed in the center platform of the maze, facing all three arms immediately before the session [58].

### Elevated plus-maze (EPM) test

Based on previous studies [18,43,62-64], alterations in anxiety-related behaviors were examined in the EPM test using a cardboard apparatus that consisted of two opposite open arms 50 × 10 cm (length and width) and two enclosed arms 50 × 10 × 30 cm (length, width, and height), positioned 50 cm from the floor. After the number of entries into open arms, the time spent on open arms (sec), and the total number of entries into arms were evaluated (5 min test periods), the percentage of entries into open arms and the percentage of time spent on open arms were calculated as parameters for the anxiety-related behaviors. The total number of entries into arms was assessed as a parameter representing locomotor activity [63]. Considering the previous data [43], the evaluations of these parameters were performed at the 2 h time point after the last NC, IM or NC-IM treatment. At the beginning of each experimental session, each mouse was placed diagonally in the center platform of the maze, facing both the open and enclosed arms [43].

### Statistical analysis

The data were subjected to two-way analysis of variance (ANOVA) for both effects of NC and/or IM and effects of the CB ligands [65]. With respect to the experiments examining the effects of NC and/or IM, a 3 (0.3 mg/kg NC, 0.8 mg/kg NC versus vehicle) × 2 (IM versus vehicle) factorial design was used for the factors NC × IM treatment. With respect to the experiments examining the effects of the CB ligands, a 4 (NC, IM, NC-IM versus vehicle) × 4 (three doses of each CB ligand versus vehicle) factorial design was used for the factors NC and/or IM treatment × treatment using each CB ligand. For pairwise comparisons, Bonferroni post-hoc tests were performed [65]. All of the comparisons were performed using a statistical software package (“Excel Statistics” from Social Survey Research Information Co. Ltd. Inc., Tokyo, Japan). P values less than 0.05 were considered to be statistically significant.

## Results

### NC- and/or IM-induced working memory-related behavioral alterations in the Y-maze test

**Figure 1:**
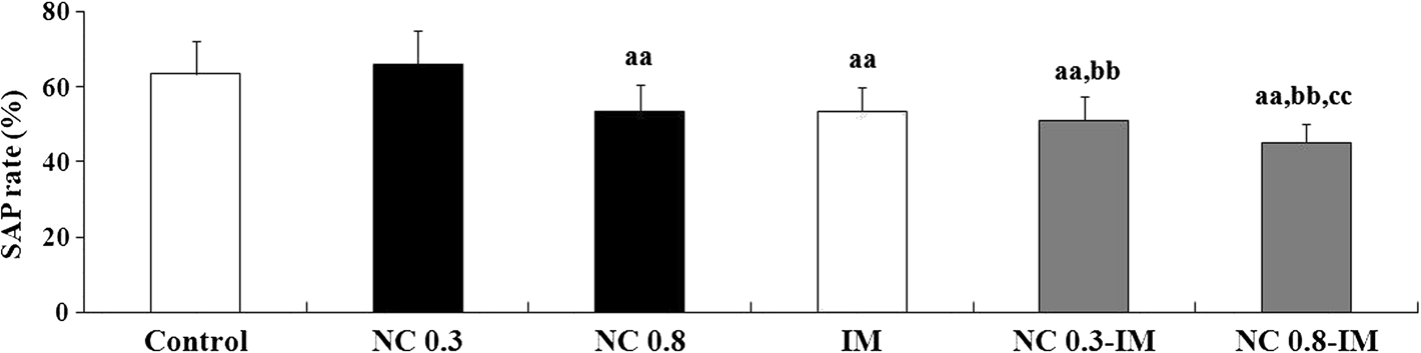
**Working memory-related behavioral alterations (SAP rate (%)) caused by repeated nicotine (NC) and/or immobilization stress (IM) in the Y-maze test.** The values at the 2 h time point after the last NC (0.3 or 0.8 mg/kg, s.c.) or IM treatment are shown as means ± S.D. (n=10 for each group). aa (p<0.01): significant attenuation as compared to the control group; bb (p<0.01): significant attenuation as compared to the NC group; cc (p<0.01): significant attenuation as compared to the IM group.

In the 0.8 mg/kg NC, IM and NC-IM groups, at the 2 h time point, behavioral alterations indicating working memory impairments, i.e. statistically significantly attenuated SAP rates (Figure 1), in spite of the absence of significant changes in the total numbers of entries into arms (Table 1), were observed in the Y-maze test. This is consistent with the results of the ANOVA revealing statistically significant main effects of NC (F(2, 54)=11.02, P<0.001) and IM (F(1, 54)=34.03, P<0.001). For the NC-IM groups, the SAP rates were significantly attenuated as compared to the NC and/or IM groups.

**Table 1:** **Total number of entries into arms in experiments examining the effects of NC and/or IM (experiments shown in Figures**1**and**2**)**

	**Y-maze test (Figure** 1**)**	**EPM test (Figure** 2**)**
Control group	47.1 ± 8.3	63.2 ± 12.4
NC 0.3 group	51.2 ± 8.4	59.1 ± 12.3
NC 0.8 group	43.7 ± 8.5	58.5 ± 11.9
IM group	44.5 ± 8.2	60.8 ± 12.1
NC 0.3-IM group	49.0 ± 8.6	56.5 ± 12.0
NC 0.8-IM group	43.2 ± 8.6	55.0 ± 11.7

### NC- and/or IM-induced anxiety-related behavioral alterations in the EPM test

**Figure 2:**
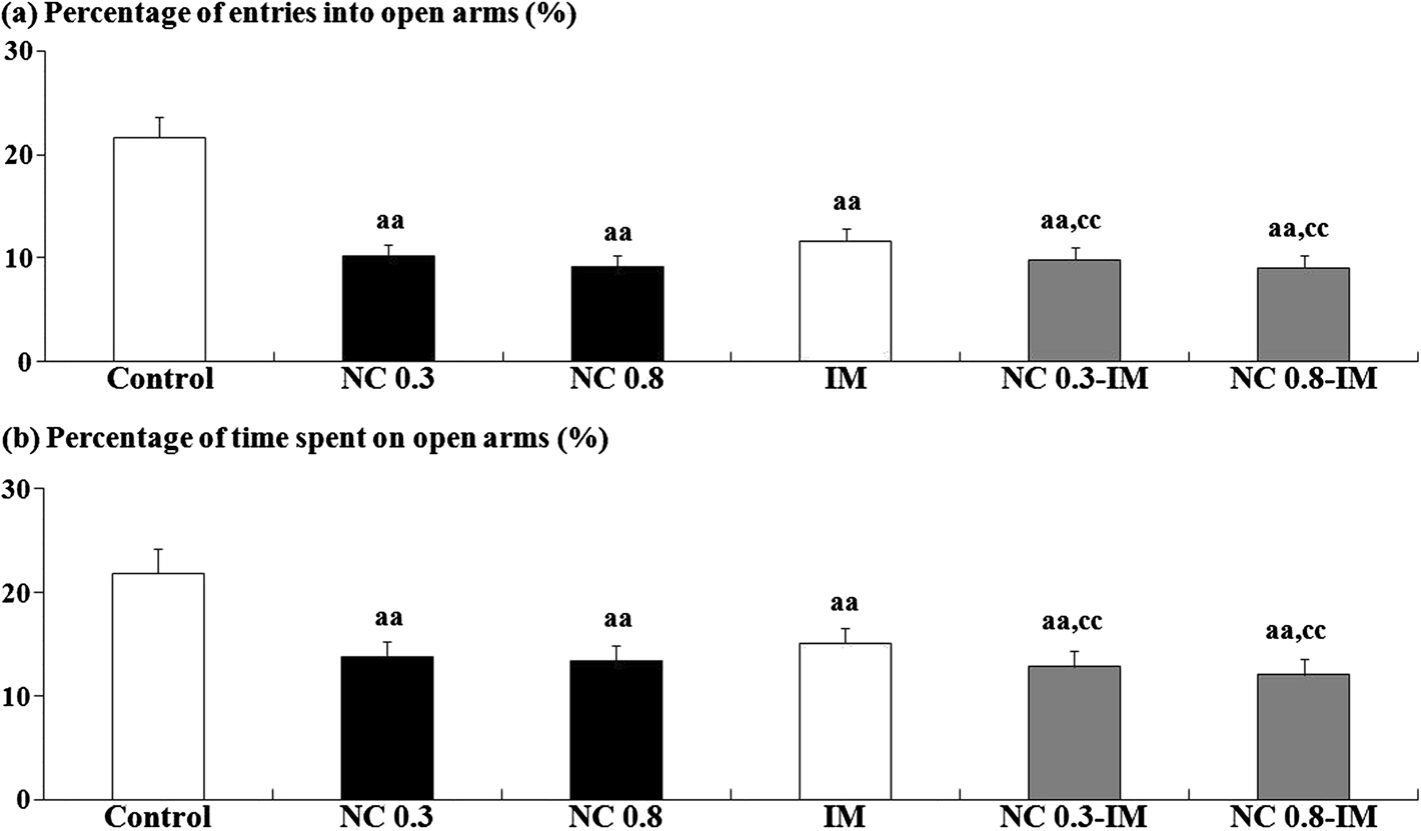
**Anxiety-related behavioral alterations caused by repeated nicotine (NC) and/or immobilization stress (IM) in the elevated plus-maze (EPM) test.** Data are presented for percentage of entries into open arms (**a**) and percentage of time spent on open arms (**b**). The values at the 2 h time point after the last NC (0.3 or 0.8 mg/kg, s.c.) or IM treatment are shown as means ± S.D. (n=10 for each group). aa (p<0.01): significant attenuation as compared to the control group; cc (p<0.01): significant attenuation as compared to the IM group.

In the NC, IM and NC-IM groups, at the 2 h time point, anxiety-like behavioral alterations, i.e. statistically significantly attenuated percentage of entries into open arms (Figure 2a) and significantly attenuated percentage of time spent on open arms (Figure 2b), in spite of the absence of significant changes in the total numbers of entries into arms (Table 1), were observed in the EPM test. This is consistent with the results of the ANOVA revealing statistically significant main effects of NC (F(2, 54)=195.21, P<0.001 for the percentage of entries into open arms and F(2, 54)=70.18, P<0.001 for the percentage of time spent on open arms) and IM (F(1, 54)=104.38, P<0.001 for the percentage of entries into open arms and F(1, 54)=46.89, P<0.001 for the percentage of time spent on open arms). For the NC-IM groups, the parameter values were significantly attenuated as compared to the IM group, which is consistent with the results of the ANOVA revealing significant interactions between the NC and IM treatments for each parameter value (F(2, 54)=91.17, P<0.001 for the percentage of entries into open arms and F(2, 54)=18.90, P<0.001 for the percentage of time spent on open arms).

### Effects of CB ligands against NC (0.8 mg/kg)- and/or IM-induced working memory-related behavioral alterations in the Y-maze test

**Figure 3:**
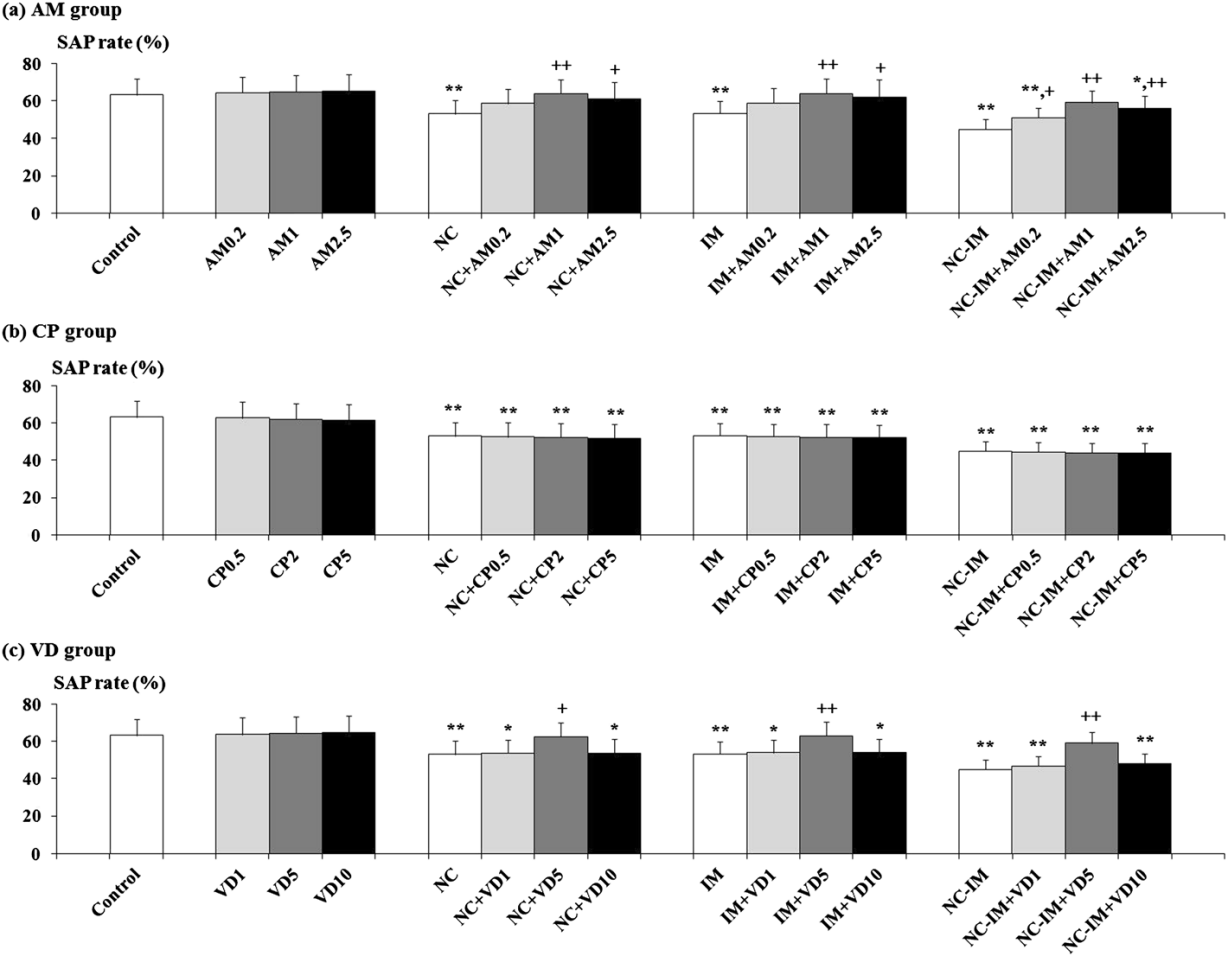
**Effects of cannabinoid receptor ligands (CB ligands) on the working memory-related behavioral alterations (SAP rate (%)) caused by repeated nicotine (NC) and/or immobilization stress (IM) in the Y-maze test.** Data of SAP rate are presented for groups of mice co-treated with AM (**a**), CP (**b**) and VD (**c**). The values at the 2 h time point after the last NC (0.8 mg/kg, s.c.) or IM treatment are shown as means ± S.D. (n=10 for each group). The abbreviations of the co-administered CB ligands with each i.p. dose (mg/kg) are noted in the text. The data for the control, NC, IM, and NC plus IM (NC-IM) groups without any CB ligand co-treatments, as well as the CB ligand-only groups, are also shown. * (p<0.05), ** (p<0.01): significant attenuation as compared to the control group; + (p<0.05), ++ (p<0.01): significant increase as compared to the NC, IM, or NC plus IM (NC-IM) group without any CB ligand co-treatments.

For the 0.8 mg/kg NC, IM and 0.8 mg/kg NC-IM groups, at the 2 h time point, statistically significant recoveries from the impairments in working memory-related behavioral alterations, i.e. recoveries from the attenuated SAP rates, in spite of the absence of significant changes in the total numbers of entries into arms (Table 2), were observed in the groups co-treated with AM (Figure 3a). This is consistent with the results of the ANOVA revealing statistically significant main effects of AM (F(3, 144)=11.20, P<0.001) for the SAP rate. Furthermore, in the groups co-treated with 5 mg/kg VD, significant recoveries from the behavioral alterations were also observed (Figure 3c), which is consistent with the results of the ANOVA revealing statistically significant main effects of VD (F(3, 144)=11.74, P<0.001) for the SAP rate. In each CB ligand-only group, no significant alterations as compared to the control group were observed for each parameter value under the present experimental conditions.

**Table 2:** **Total number of entries into arms in experiments examining the effects of CB ligands in the Y-maze test (experiments shown in Figure**3**)**

**(a) AM group**	**Control**	**NC**	**IM**	**NC-IM**
Control group	47.1 ± 8.3	43.7 ± 8.5	44.5 ± 8.2	43.2 ± 8.6
AM 0.2 group	46.9 ± 8.3	44.0 ± 8.6	45.1 ± 8.2	43.7 ± 8.6
AM 1 group	46.6 ± 8.3	44.5 ± 8.5	45.5 ± 8.3	44.1 ± 8.6
AM 2.5 group	46.2 ± 8.3	45.0 ± 8.6	46.0 ± 8.3	44.6 ± 8.6
**(b) CP group**	**Control**	**NC**	**IM**	**NC-IM**
Control group	47.1 ± 8.3	43.7 ± 8.5	44.5 ± 8.2	43.2 ± 8.6
CP 0.5 group	46.8 ± 8.3	43.5 ± 8.6	44.2 ± 8.4	43.0 ± 8.7
CP 2 group	46.5 ± 8.4	43.4 ± 8.6	44.0 ± 8.5	42.8 ± 8.8
CP 5 group	46.1 ± 8.4	43.2 ± 8.7	43.7 ± 8.5	42.6 ± 8.9
**(c) VD group**	**Control**	**NC**	**IM**	**NC-IM**
Control group	47.1 ± 8.3	43.7 ± 8.5	44.5 ± 8.2	43.2 ± 8.6
VD 1 group	46.8 ± 8.4	43.9 ± 8.6	44.8 ± 8.3	43.5 ± 8.7
VD 5 group	46.5 ± 8.4	44.2 ± 8.7	45.1 ± 8.4	43.7 ± 8.8
VD 10 group	46.2 ± 8.5	44.4 ± 8.8	45.4 ± 8.5	43.9 ± 8.9

### Effects of CB ligands against NC (0.8 mg/kg)- and/or IM-induced anxiety-related behavioral alterations in the EPM test

**Figure 4:**
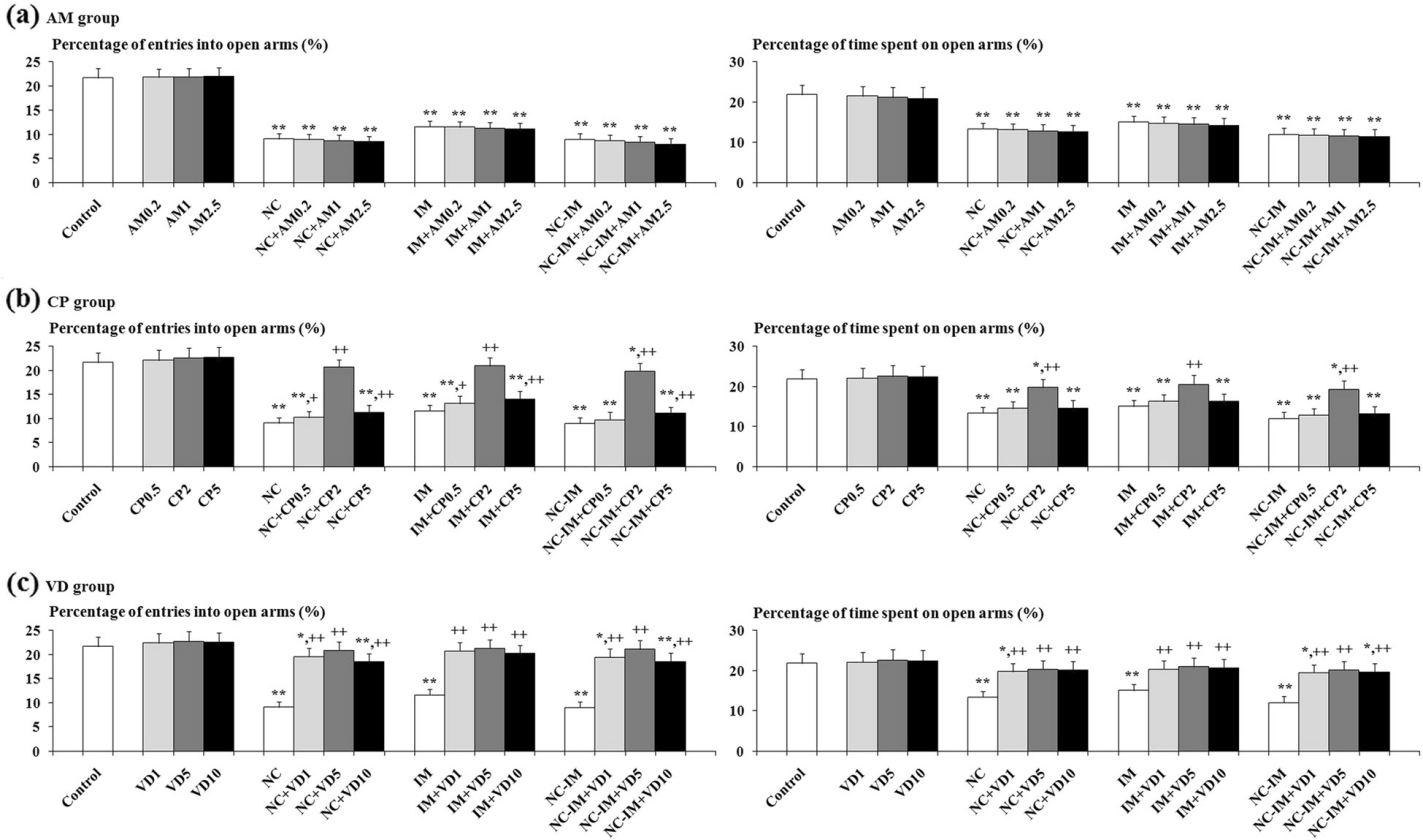
**Effects of cannabinoid receptor ligands (CB ligands) on the anxiety-related behavioral alterations caused by repeated nicotine (NC) and/or immobilization stress (IM) in the elevated plus-maze (EPM) test.** Data of percentages of entries into open arms and time spent on open arms are presented for groups of mice co-treated with AM (**a**), CP (**b**) and VD (**c**). The values at the 2 h time point after the last NC (0.8 mg/kg, s.c.) or IM treatment are shown as means ± S.D. (n=10 for each group). The abbreviations of the co-administered CB ligands with each i.p. dose (mg/kg) are noted in the text. The data for the control, NC, IM, and NC plus IM (NC-IM) groups without any CB ligand co-treatments, as well as the CB ligand-only groups, are also shown. * (p<0.05), ** (p<0.01): significant attenuation as compared to the control group; + (p<0.05), ++ (p<0.01): significant increase as compared to the NC, IM, or NC plus IM (NC-IM) group without any CB ligand co-treatments.

For the 0.8 mg/kg NC, IM and 0.8 mg/kg NC-IM groups, at the 2 h time point, statistically significant recoveries from the anxiety-like behavioral alterations, i.e. recoveries from both attenuated percentage of entries into open arms and attenuated percentage of time spent on open arms, in spite of the absence of significant changes in the total numbers of entries into arms (Table 3), were observed in the groups co-treated with VD (1–10 mg/kg) (Figure 4c). This is consistent with the results of the ANOVA revealing statistically significant main effects of VD (F(3, 144)=205.84, P<0.001 for the percentage of entries into open arms and F(3, 144)=58.29, P<0.001 for the percentage of time spent on open arms) and significant interactions of the VD versus NC and/or IM treatments (F(9, 144)=18.88, P<0.001 for the percentage of entries into open arms and F(9, 144)=5.58, P<0.001 for the percentage of time spent on open arms). Furthermore, in the groups co-treated with 2 mg/kg CP, significant recoveries in both entries into and time spent on open arms were also observed (Figure 4b), which is consistent with the results of the ANOVA revealing statistically significant main effects of CP (F(3, 144)=206.08, P<0.001 for the percentage of entries into open arms and F(3, 144)=47.32, P<0.001 for the percentage of time spent on open arms) and significant interactions of the CP versus NC and/or IM treatments (F(9, 144)=20.61, P<0.001 for the percentage of entries into open arms and F(9, 144)=4.50, P<0.001 for the percentage of time spent on open arms). In each CB ligand-only group, no significant alterations as compared to the control group were observed for each parameter value under the present experimental conditions.

**Table 3:** **Total number of entries into arms in experiments examining the effects of CB ligands in the EPM test (experiments shown in Figure**4**)**

**(a) AM group**	**Control**	**NC**	**IM**	**NC-IM**
Control group	63.2 ± 12.4	58.5 ± 11.9	60.8 ± 12.1	55.0 ± 11.7
AM 0.2 group	62.7 ± 12.6	58.9 ± 12.0	61.1 ± 12.3	55.5 ± 12.1
AM 1 group	62.4 ± 12.9	59.4 ± 12.2	61.5 ± 12.4	56.2 ± 12.4
AM 2.5 group	61.9 ± 13.1	59.9 ± 12.6	61.7 ± 12.7	57.5 ± 12.7
**(b) CP group**	**Control**	**NC**	**IM**	**NC-IM**
Control group	63.2 ± 12.4	58.5 ± 11.9	60.8 ± 12.1	55.0 ± 11.7
CP 0.5 group	62.6 ± 12.7	58.3 ± 12.0	60.6 ± 12.3	54.8 ± 12.1
CP 2 group	62.1 ± 12.8	58.0 ± 12.1	60.3 ± 12.4	54.7 ± 12.3
CP 5 group	61.5 ± 13.0	57.8 ± 12.2	60.0 ± 12.5	54.6 ± 12.5
**(c) VD group**	**Control**	**NC**	**IM**	**NC-IM**
Control group	63.2 ± 12.4	58.5 ± 11.9	60.8 ± 12.1	55.0 ± 11.7
VD 1 group	62.6 ± 12.6	58.8 ± 12.1	61.0 ± 12.4	54.9 ± 12.2
VD 5 group	62.3 ± 12.8	59.2 ± 12.2	61.3 ± 12.4	55.9 ± 12.4
VD 10 group	61.7 ± 13.0	59.5 ± 12.6	61.5 ± 12.7	56.9 ± 12.7

## Discussion

### NC- and/or IM-induced working memory- and anxiety-related behavioral alterations

In the NC group using repeated treatments of 0.8 mg/kg NC, as well as in the IM group, behavioral alterations suggestive of working memory impairments were observed in the Y-maze test (attenuated SAP rate) (Figure 1). However, in the NC group using repeated treatments of 0.3 mg/kg NC, in spite of the appearance of anxiety-like behavioral alterations similar to the IM group (Figure 2), the absence of overt working memory-related behavioral alterations (a slightly increased SAP rate) was observed in the Y-maze test (Figure 1). Previous studies have shown that brain serotonergic and cholinergic systems play crucial roles in mediating anxiety-related behavioral responses [66-68]. In addition to these systems, several neuroendocrine responses (e.g. secretion of corticosterone, norepinephrine, etc.) have been reported to participate in the control of anxiety-like behaviors [69,70]. Similar modifications in these responses were observed between NC and IM [71], and may contribute to their anxiogenic-like effects in the present study (Figure 2). On the other hand, the NC-induced impairments in working memory, unlike the IM-induced impairments, occurred with a limited range of doses (Figure 1). It has been reported that modifications in working memory (both ameliorations and impairments) can occur due to even minuscule changes in prefrontal dopamine (DA) levels [72]. Therefore, the working memory-related behaviors in the NC groups may be correlated with characteristic but subtle alterations in nAChR-mediated prefrontal DA release, which was controlled by specific nAChR subtypes (e.g. alpha7 nAChRs) [73]. In addition to DA release, the release of other neurotransmitters such as glutamate has been implicated in the NC-induced working memory processes [74].

With respect to the interactions between NC and IM in the NC-IM group, both NC and IM enhanced each other’s effects on the working memory- and anxiety-related behavioral alterations in the Y-maze and EPM tests. Although the relationship between stressors such as IM and NC remains controversial as mentioned above (i.e. “antistress” effects of NC have also been reported depending on the conditions), synergistic effects like those observed in previous studies [34-36] were provided by the NC plus IM treatment in the present experimental model. An augmented increase in secreted hypothalamic-pituitary-adrenal (HPA) hormones and/or immediate early gene expression was demonstrated in those studies [35,36]. Furthermore, the involvement of these molecular changes has also been reported for working memory- and anxiety-related behaviors [75-78]. However, further analyses are needed to elucidate the mechanisms underlying these complex interactions between NC and IM.

### Effects of cannabinoid (CB) ligands

Consistent with the previous studies described above [18,43,49,50,52], the NC- and/or IM-induced working memory impairment-like behaviors were antagonized by the CB1 antagonist AM (Figure 3a), and the anxiety-like behaviors were antagonized by the CB agonist CP (Figure 4b). Furthermore, VD, a mixed CB1 ligand with partial agonist plus antagonist activities [79], provided recovering effects against the impaired behaviors related to both working memory and anxiety (Figure 3c, 4c). Similarities in some neuronal responses such as prefrontal DA responses have been observed in the immunohistochemical studies between the effects of NC, stressors, and CB1 agonists [21,80-82]. These similarities may result in the CB1 antagonist-induced recoveries in the working memory-related behavioral alterations. On the other hand, against the NC- and/or IM-induced anxiety-related behavioral alterations, only the CB1 ligands acting at least partially as agonists exerted any anxiolytic-like effects. Although the mechanisms underlying these discrepancies in the effects of CB ligands have not been elucidated, anxiolytic-like effects of CB agonists have been shown under several different conditions [83,84]. Furthermore, certain doses of CB1 agonists have been reported to be able to activate the neurotransmission systems related to anti-anxiety (e.g. GABAergic and serotonergic systems) at the molecular level [53,83,84]. Therefore, it could be predicted that the antagonistic effects against the anxiety-like behaviors were provided at least indirectly by way of agonistic activity on CB1 receptors.

Against both working memory- and anxiety-related behavioral alterations, the CB1 partial agonist/antagonist VD exerted some recovering effects (Figure 3c, 4c). These recovering effects were observed equally in the NC, IM and NC-IM groups. For the behavioral alterations related to working memory impairments, the recovering effects of VD did not exceed those of the CB1 antagonist AM, which could be predicted considering the partial CB1 agonistic effects of VD. On the other hand, for the behavioral alterations related to anxiety, the recovering effects of VD exceeded those of the CB agonist CP. These results could not be predicted considering the above-mentioned anxiolytic-like effects of the CB1 agonists without any antagonistic effects. However, contrary to the present results, there are experimental data showing anxiogenic-like or anti-anxiolytic effects provided by high doses of CB agonists including CP [53,54,85]. The involvement of an abnormal release of anxiety-related neurotransmitters has been reported for those effects [53,54]. Unlike CP, VD possesses CB1 antagonistic potential for counteracting anti-anxiolytic effects as an agonist (high doses), and thus may function as an effective anxiolytic-like ligand. Furthermore, recent studies suggest the possibility that CB2 receptors, the CB receptors initially defined as peripheral receptors, may contribute to anti-anxiolytic effects [86,87]. CB2 receptors are distributed over several regions of the central nervous system [87] and the non-selective CB agonist CP seems to provide agonistic effects as well as against CB1 receptors. With respect to the effects of AM and VD, agonistic effects on the GPR55 receptor subtype, a newly-identified G protein coupled CB receptor subtype, have also been reported [88-91]. Although the behavioral roles of GPR55 receptors have not been investigated, it is possible that GPR55 distributed in the brain [89] may participate in cognitive processes such as working memory. Furthermore, in vitro studies demonstrated that VD acted as a partial agonist on GPR55 receptors and provided antagonistic effects at high concentrations [91], whereas only full agonistic effects have been reported for AM [88-90]. On the other hand, on CB1 receptors, both VD and AM provided antagonistic effects at high concentrations [79,89]. These characteristic effects of the partial agonist/antagonist VD as a GPR55 antagonist at high concentrations may be correlated with its limited and non-dose-dependent ameliorating effects against working memory impairments, i.e. only 5 mg/kg VD, but not a lower (1 mg/kg) or higher (10 mg/kg) dose, was effective. In addition to GPR55, the existence of other yet-to-be-cloned CB receptors has been suggested in memory-related brain regions such as the hippocampus [92]. There may be some contributions of these receptors to the AM- and VD-derived attenuating effects against the NC- and/or IM-induced working memory impairments.

## Conclusions

The present study demonstrated working memory impairment- and anxiety-like behaviors induced by NC, IM and NC-IM treatments in mice. Mutual synergistic effects for NC plus IM were observed for both types of behavioral alterations. In the present study, the involvement of endocannabinoid system was also shown in the processes of working memory and anxiety. However, between the working memory- and anxiety-related behavioral alterations, discrepancies in the types of effective CB ligands were observed: the CB1 antagonist AM was the most effective against the working memory impairment-related behaviors, whereas the CB1 partial agonist/antagonist VD was the most effective against the anxiety-related behaviors. Since the presence of new CB receptor subtypes such as GPR55 receptors has been clarified recently and the interactions with each CB ligand have been suggested, further research into the therapeutic contributions of each CB receptor subtype is expected.

## Competing interests

The author declares that there are no potential competing interests.

## Authors’ contributions

TH designed the study, carried out all experiments and statistical analyses, and prepared the manuscript.

## References

[B1] Zarocostas J WHO report warns deaths from tobacco could rise beyond eight million a year by 2030 BMJ 2008 336 299 18258960 10.1136/bmj.39483.532361.DB PMC2234512

[B2] WHO report on the global tobacco epidemic 2011 [http://www.who.int/tobacco/global_report/2011/en/]

[B3] Heishman SJ Behavioral and cognitive effects of smoking: relationship to nicotine addiction Nicotine Tob Res 1999 1 Suppl 2 143 147 11768172 10.1080/14622299050011971

[B4] Anstey KJ von Sanden C Salim A O'Kearney R Smoking as a risk factor for dementia and cognitive decline: a meta-analysis of prospective studies Am J Epidemiol 2007 166 367 378 10.1093/aje/kwm116 17573335

[B5] Llewellyn DJ Lang IA Langa KM Naughton F Matthews FE Exposure to secondhand smoke and cognitive impairment in non-smokers: national cross sectional study with cotinine measurement BMJ 2009 338 b462 10.1136/bmj.b462 19213767 PMC2643443

[B6] Khan ZU Muly EC Molecular mechanisms of working memory Behav Brain Res 2011 219 329 341 10.1016/j.bbr.2010.12.039 21232555

[B7] Xu J Mendrek A Cohen MS Monterosso J Rodriguez P Simon SL Brody A Jarvik M Domier CP Olmstead R Ernst M London ED Brain activity in cigarette smokers performing a working memory task: effect of smoking abstinence Biol Psychiatry 2005 58 143 150 10.1016/j.biopsych.2005.03.028 16038685 PMC2773671

[B8] Malin DH Goyarzu P Rodent models of nicotine withdrawal syndrome Handb Exp Pharmacol 2009 192 401 434 10.1007/978-3-540-69248-5_14 19184657

[B9] Park S Knopick C McGurk S Meltzer HY Nicotine impairs spatial working memory while leaving spatial attention intact Neuropsychopharmacology 2000 22 200 209 10.1016/S0893-133X(99)00098-6 10649832

[B10] Jacobsen LK Krystal JH Mencl WE Westerveld M Frost SJ Pugh KR Effects of smoking and smoking abstinence on cognition in adolescent tobacco smokers Biol Psychiatry 2005 57 56 66 10.1016/j.biopsych.2004.10.022 15607301

[B11] Swan GE Lessov-Schlaggar CN The effects of tobacco smoke and nicotine on cognition and the brain Neuropsychol Rev 2007 17 259 273 10.1007/s11065-007-9035-9 17690985

[B12] Toledano A Alvarez MI Toledano-Díaz A Diversity and variability of the effects of nicotine on different cortical regions of the brain - therapeutic and toxicological implications Cent Nerv Syst Agents Med Chem 2010 10 180 206 10.2174/1871524911006030180 20528766

[B13] Foulds J Stapleton J Swettenham J Bell N McSorley K Russell MA Cognitive performance effects of subcutaneous nicotine in smokers and never-smokers Psychopharmacology (Berl) 1996 127 31 38 10.1007/BF02805972 8880941

[B14] Phillips S Fox P An investigation into the effects of nicotine gum on short-term memory Psychopharmacology (Berl) 1998 140 429 433 10.1007/s002130050786 9888618

[B15] Rezvani AH Levin ED Cognitive effects of nicotine Biol Psychiatry 2001 49 258 267 10.1016/S0006-3223(00)01094-5 11230877

[B16] Ernst M Heishman SJ Spurgeon L London ED Smoking history and nicotine effects on cognitive performance Neuropsychopharmacology 2001 25 313 319 10.1016/S0893-133X(01)00257-3 11522460

[B17] Kleykamp BA Jennings JM Blank MD Eissenberg T The effects of nicotine on attention and working memory in never-smokers Psychol Addict Behav 2005 19 433 438 16366815 10.1037/0893-164X.19.4.433

[B18] Balerio GN Aso E Maldonado R Role of the cannabinoid system in the effects induced by nicotine on anxiety-like behaviour in mice Psychopharmacology (Berl) 2006 184 504 513 10.1007/s00213-005-0251-9 16416159

[B19] Caldarone BJ King SL Picciotto MR Sex differences in anxiety-like behavior and locomotor activity following chronic nicotine exposure in mice Neurosci Lett 2008 439 187 191 10.1016/j.neulet.2008.05.023 18524488 PMC2491450

[B20] Kupferschmidt DA Funk D Erb S Lê AD Age-related effects of acute nicotine on behavioural and neuronal measures of anxiety Behav Brain Res 2010 213 288 292 10.1016/j.bbr.2010.05.022 20546793

[B21] Mizoguchi K Yuzurihara M Ishige A Sasaki H Chui DH Tabira T Chronic stress induces impairment of spatial working memory because of prefrontal dopaminergic dysfunction J Neurosci 2000 20 1568 1574 10662846 10.1523/JNEUROSCI.20-04-01568.2000 PMC6772382

[B22] Shansky RM Rubinow K Brennan A Arnsten AF The effects of sex and hormonal status on restraint-stress-induced working memory impairment Behav Brain Funct 2006 2 8 10.1186/1744-9081-2-8 16522198 PMC1420310

[B23] Schoofs D Preuss D Wolf OT Psychosocial stress induces working memory impairments in an n-back paradigm Psychoneuroendocrinology 2008 33 643 653 10.1016/j.psyneuen.2008.02.004 18359168

[B24] MacNeil G Sela Y McIntosh J Zacharko RM Anxiogenic behavior in the light–dark paradigm follwoing intraventricular administration of cholecystokinin-8S, restraint stress, or uncontrollable footshock in the CD-1 mouse Pharmacol Biochem Behav 1997 58 737 746 10.1016/S0091-3057(97)00037-3 9329067

[B25] Chotiwat C Harris RB Increased anxiety-like behavior during the post-stress period in mice exposed to repeated restraint stress Horm Behav 2006 50 489 495 10.1016/j.yhbeh.2006.06.007 16870191

[B26] Middleton LS Cass WA Dwoskin LP Nicotinic receptor modulation of dopamine transporter function in rat striatum and medial prefrontal cortex J Pharmacol Exp Ther 2004 308 367 377 14563785 10.1124/jpet.103.055335

[B27] Singer S Rossi S Verzosa S Hashim A Lonow R Cooper T Sershen H Lajtha A Nicotine-induced changes in neurotransmitter levels in brain areas associated with cognitive function Neurochem Res 2004 29 1779 1792 15453274 10.1023/b:nere.0000035814.45494.15

[B28] Wall PM Messier C Concurrent modulation of anxiety and memory Behav Brain Res 2000 109 229 241 10.1016/S0166-4328(99)00177-1 10762693

[B29] Clinton SM Sucharski IL Finlay JM Desipramine attenuates working memory impairments induced by partial loss of catecholamines in the rat medial prefrontal cortex Psychopharmacology (Berl) 2006 183 404 412 10.1007/s00213-005-0221-2 16307295

[B30] Blanco E Castilla-Ortega E Miranda R Begega A Aguirre JA Arias JL Santín LJ Effects of medial prefrontal cortex lesions on anxiety-like behaviour in restrained and non-restrained rats Behav Brain Res 2009 201 338 342 10.1016/j.bbr.2009.03.001 19428654

[B31] Goldwater DS Pavlides C Hunter RG Bloss EB Hof PR McEwen BS Morrison JH Structural and functional alterations to rat medial prefrontal cortex following chronic restraint stress and recovery Neuroscience 2009 164 798 808 10.1016/j.neuroscience.2009.08.053 19723561 PMC2762025

[B32] Parrott AC Does cigarette smoking cause stress? Am Psychol 1999 54 817 820 10540594 10.1037//0003-066x.54.10.817

[B33] Parrott AC Kaye FJ Daily uplifts, hassles, stresses and cognitive failures: in cigarette smokers, abstaining smokers, and non-smokers Behav Pharmacol 1999 10 639 646 10.1097/00008877-199911000-00010 10780505

[B34] Kita T Okamoto M Kubo K Tanaka T Nakashima T Enhancement of sensitization to nicotine-induced ambulatory stimulation by psychological stress in rats Prog Neuropsychopharmacol Biol Psychiatry 1999 23 893 903 10.1016/S0278-5846(99)00033-0 10509382

[B35] Lutfy K Brown MC Nerio N Aimiuwu O Tran B Anghel A Friedman TC Repeated stress alters the ability of nicotine to activate the hypothalamic-pituitary-adrenal axis J Neurochem 2006 99 1321 1327 10.1111/j.1471-4159.2006.04217.x 17064351

[B36] Schiltz CA Kelley AE Landry CF Acute stress and nicotine cues interact to unveil locomotor arousal and activity-dependent gene expression in the prefrontal cortex Biol Psychiatry 2007 61 127 135 10.1016/j.biopsych.2006.03.002 16631128 PMC1698504

[B37] Childs E de Wit H Hormonal, cardiovascular, and subjective responses to acute stress in smokers Psychopharmacology (Berl) 2009 203 1 12 10.1007/s00213-008-1359-5 18936915 PMC2727744

[B38] Kotlyar M Drone D Thuras P Hatsukami DK Brauer L Adson DE al'Absi M Effect of stress and bupropion on craving, withdrawal symptoms, and mood in smokers Nicotine Tob Res 2011 13 492 497 10.1093/ntr/ntr011 21378081 PMC3103714

[B39] Minowa K Pawlak R Takada Y Takada A Nicotine attenuates stress-induced changes in plasma amino acid concentrations and locomotor activity in rats Brain Res Bull 2000 51 83 88 10.1016/S0361-9230(99)00207-5 10654585

[B40] Hsu HR Chen TY Chan MH Chen HH Acute effects of nicotine on restraint stress-induced anxiety-like behavior, c-Fos expression, and corticosterone release in mice Eur J Pharmacol 2007 566 124 131 10.1016/j.ejphar.2007.03.040 17459372

[B41] Andreasen JT Henningsen K Bate S Christiansen S Wiborg O Nicotine reverses anhedonic-like response and cognitive impairment in the rat chronic mild stress model of depression: comparison with sertraline J Psychopharmacol 2011 25 1134 1141 10.1177/0269881110391831 21169388

[B42] Aleisa AM Alzoubi KH Gerges NZ Alkadhi KA Nicotine blocks stress-induced impairment of spatial memory and long-term potentiation of the hippocampal CA1 region Int J Neuropsychopharmacol 2006 9 417 426 10.1017/S1461145705005912 16316479

[B43] Hayase T Chronologically overlapping occurrences of nicotine-induced anxiety- and depression-related behavioral symptoms: effects of anxiolytic and cannabinoid drugs BMC Neurosci 2007 8 76 10.1186/1471-2202-8-76 17877812 PMC2075518

[B44] Hayase T Depression-related anhedonic behaviors caused by immobilization stress: a comparison with nicotine-induced depression-like behavioral alterations and effects of nicotine and/or “antidepressant” drugs J Toxicol Sci 2011 36 31 41 10.2131/jts.36.31 21297339

[B45] Riebe CJ Wotjak CT Endocannabinoids and stress Stress 2011 14 384 397 21663537 10.3109/10253890.2011.586753

[B46] Castañé A Valjent E Ledent C Parmentier M Maldonado R Valverde O Lack of CB1 cannabinoid receptors modifies nicotine behavioural responses, but not nicotine abstinence Neuropharmacology 2002 43 857 867 10.1016/S0028-3908(02)00118-1 12384171

[B47] Picciotto MR Caldarone BJ King SL Zachariou V Nicotinic receptors in the brain. Links between molecular biology and behavior. Neuropsychopharmacology 2000 22 451 465 10.1016/S0893-133X(99)00146-3 10731620

[B48] Viveros MP Marco EM Llorente R Lamota L The role of the hippocampus in mediating emotional responses to nicotine and cannabinoids: a possible neural substrate for functional interactions Behav Pharmacol 2007 18 375 389 10.1097/FBP.0b013e3282d28fb4 17762508

[B49] Riedel G Davies SN Cannabinoid function in learning, memory and plasticity Handb Exp Pharmacol 2005 168 445 477 10.1007/3-540-26573-2_15 16596784

[B50] Marsicano G Lafenêtre P Roles of the endocannabinoid system in learning and memory Curr Top Behav Neurosci 2009 1 201 230 10.1007/978-3-540-88955-7_8 21104385

[B51] Biala G Kruk M Cannabinoid receptor ligands suppress memory-related effects of nicotine in the elevated plus maze test in mice Behav Brain Res 2008 192 198 202 10.1016/j.bbr.2008.04.004 18501975

[B52] Mateos B Borcel E Loriga R Luesu W Bini V Llorente R Castelli MP Viveros MP Adolescent exposure to nicotine and/or the cannabinoid agonist CP 55,940 induces gender-dependent long-lasting memory impairments and changes in brain nicotinic and CB(1) cannabinoid receptors J Psychopharmacol 2011 25 1676 1690 10.1177/0269881110370503 20562169

[B53] Viveros MP Marco EM File SE Endocannabinoid system and stress and anxiety responses Pharmacol Biochem Behav 2005 81 331 342 10.1016/j.pbb.2005.01.029 15927244

[B54] Moreira FA Wotjak CT Cannabinoids and anxiety Curr Top Behav Neurosci 2010 2 429 450 21309120 10.1007/7854_2009_16

[B55] Hayase T Yamamoto Y Yamamoto K Stress-related behavioral alterations accompanying cocaine toxicity: the effects of mixed opioid drugs Nihon Arukoru Yakubutsu Igakkai Zasshi 2000 35 402 414 11197874

[B56] Committee on Animal Research of Kyoto University Faculty of Medicine Guidelines for Animal Experiments of Kyoto University Faculty of Medicine [http://www.kyoto-u.ac.jp/uni_int/kitei/reiki_honbun/w002RG00001169.html] (Japanese)

[B57] Armario A Gil M Marti J Pol O Balasch J Influence of various acute stressors on the activity of adult male rats in a holeboard and in the forced swim test Pharmacol Biochem Behav 1991 39 373 377 10.1016/0091-3057(91)90194-7 1946578

[B58] Hayase T Memory-related and anxiogenic effects of nicotine: roles of cannabinoid receptors Nihon Arukoru Yakubutsu Igakkai Zasshi 2009 44 440 441 (Japanese)

[B59] Yoshida T Sakane N Umekawa T Kondo M Effect of nicotine on sympathetic nervous system activity of mice subjected to immobilization stress Physiol Behav 1994 55 53 57 10.1016/0031-9384(94)90009-4 8140174

[B60] Sarter M Bodewitz G Stephens DN Attenuation of scopolamine-induced impairment of spontaneous alteration behaviour by antagonist but not inverse agonist and agonist beta-carbolines Psychopharmacology (Berl) 1988 94 491 495 10.1007/BF00212843 2836875

[B61] Parada-Turska J Turski WA Excitatory amino acid antagonists and memory: effect of drugs acting at N-methyl-D-aspartate receptors in learning and memory tasks Neuropharmacology 1990 29 1111 1116 10.1016/0028-3908(90)90034-O 2149871

[B62] Pellow S Chopin P File SE Briley M Validation of open:closed arm entries in an elevated plus-maze as a measure of anxiety in the rat J Neurosci Methods 1985 14 149 167 10.1016/0165-0270(85)90031-7 2864480

[B63] File SE Aranko K Sodium valproate and chlordiazepoxide in the elevated plus-maze test of anxiety in the rat Neuropsychobiology 1988 20 82 86 10.1159/000118478 3151011

[B64] Prior H Schwegler H Marashi V Sachser N Exploration, emotionality, and hippocampal mossy fibers in nonaggressive AB/Gat and congenic highly aggressive mice Hippocampus 2004 14 135 140 10.1002/hipo.10166 15058491

[B65] Alves SH Pinheiro G Motta V Landeira-Fernandez J Cruz AP Anxiogenic effects in the rat elevated plus-maze of 5-HT(2C) agonists into ventral but not dorsal hippocampus Behav Pharmacol 2004 15 37 43 10.1097/00008877-200402000-00005 15075625

[B66] File SE Kenny PJ Cheeta S The role of the dorsal hippocampal serotonergic and cholinergic systems in the modulation of anxiety Pharmacol Biochem Behav 2000 66 65 72 10.1016/S0091-3057(00)00198-2 10837844

[B67] Seth P Cheeta S Tucci S File SE Nicotinic–serotonergic interactions in brain and behaviour Pharmacol Biochem Behav 2002 71 795 805 10.1016/S0091-3057(01)00715-8 11888570

[B68] Hale MW Shekhar A Lowry CA Stress-related serotonergic systems: implications for symptomatology of anxiety and affective disorders Cell Mol Neurobiol 2012 32 695 708 10.1007/s10571-012-9827-1 22484834 PMC3378822

[B69] Dallman MF Akana SF Strack AM Scribner KS Pecoraro N La Fleur SE Houshyar H Gomez F Chronic stress-induced effects of corticosterone on brain: direct and indirect Ann N Y Acad Sci 2004 1018 141 150 10.1196/annals.1296.017 15240363

[B70] Morilak DA Barrera G Echevarria DJ Garcia AS Hernandez A Ma S Petre CO Role of brain norepinephrine in the behavioral response to stress Prog Neuropsychopharmacol Biol Psychiatry 2005 29 1214 1224 10.1016/j.pnpbp.2005.08.007 16226365

[B71] Morse DE Neuroendocrine responses to nicotine and stress: enhancement of peripheral stress responses by the administration of nicotine Psychopharmacology (Berl) 1989 98 539 543 10.1007/BF00441956 2505296

[B72] Cools R D'Esposito M Inverted-U-shaped dopamine actions on human working memory and cognitive control Biol Psychiatry 2011 69 113 125 10.1016/j.biopsych.2010.04.030 21531388 PMC3111448

[B73] Livingstone PD Srinivasan J Kew JN Dawson LA Gotti C Moretti M Shoaib M Wonnacott S alpha7 and non-alpha7 nicotinic acetylcholine receptors modulate dopamine release in vitro and in vivo in the rat prefrontal cortex Eur J Neurosci 2009 29 539 550 10.1111/j.1460-9568.2009.06613.x 19187266

[B74] Timofeeva OA Levin ED Glutamate and nicotinic receptor interactions in working memory: importance for the cognitive impairment of schizophrenia Neuroscience 2011 195 21 36 21884762 10.1016/j.neuroscience.2011.08.038

[B75] Issa AM Rowe W Gauthier S Meaney MJ Hypothalamic-pituitary-adrenal activity in aged, cognitively impaired and cognitively unimpaired rats J Neurosci 1990 10 3247 3254 2170594 10.1523/JNEUROSCI.10-10-03247.1990 PMC6570181

[B76] Weiss IC Pryce CR Jongen-Rêlo AL Nanz-Bahr NI Feldon J Effect of social isolation on stress-related behavioural and neuroendocrine state in the rat Behav Brain Res 2004 152 279 295 10.1016/j.bbr.2003.10.015 15196796

[B77] Troakes C Ingram CD Anxiety behaviour of the male rat on the elevated plus maze: associated regional increase in c-fos mRNA expression and modulation by early maternal separation Stress 2009 12 362 369 10.1080/10253890802506391 19051121

[B78] Matsuo N Yamasaki N Ohira K Takao K Toyama K Eguchi M Yamaguchi S Miyakawa T Neural activity changes underlying the working memory deficit in alpha-CaMKII heterozygous knockout mice Front Behav Neurosci 2009 3 20 19750198 10.3389/neuro.08.020.2009 PMC2741293

[B79] Porter AC Sauer JM Knierman MD Becker GW Berna MJ Bao J Nomikos GG Carter P Bymaster FP Leese AB Felder CC Characterization of a novel endocannabinoid, virodhamine, with antagonist activity at the CB1 receptor J Pharmacol Exp Ther 2002 301 1020 1024 10.1124/jpet.301.3.1020 12023533

[B80] George TP Verrico CD Roth RH Effects of repeated nicotine pre-treatment on mesoprefrontal dopaminergic and behavioral responses to acute footshock stress Brain Res 1998 801 36 49 10.1016/S0006-8993(98)00537-X 9729261

[B81] Verrico CD Jentsch JD Roth RH Persistent and anatomically selective reduction in prefrontal cortical dopamine metabolism after repeated, intermittent cannabinoid administration to rats Synapse 2003 49 61 66 10.1002/syn.10215 12710016

[B82] Viveros MP Marco EM File SE Nicotine and cannabinoids: parallels, contrasts and interactions Neurosci Biobehav Rev 2006 30 1161 1181 10.1016/j.neubiorev.2006.08.002 17049986

[B83] Braida D Limonta V Malabarba L Zani A Sala M 5-HT1A receptors are involved in the anxiolytic effect of Delta9-tetrahydrocannabinol and AM 404, the anandamide transport inhibitor, in Sprague–Dawley rats Eur J Pharmacol 2007 555 156 163 10.1016/j.ejphar.2006.10.038 17116299

[B84] Haller J Mátyás F Soproni K Varga B Barsy B Németh B Mikics E Freund TF Hájos N Correlated species differences in the effects of cannabinoid ligands on anxiety and on GABAergic and glutamatergic synaptic transmission Eur J Neurosci 2007 25 2445 2456 10.1111/j.1460-9568.2007.05476.x 17445240 PMC1890583

[B85] Genn RF Tucci S Marco EM Viveros MP File SE Unconditioned and conditioned anxiogenic effects of the cannabinoid receptor agonist CP 55,940 in the social interaction test Pharmacol Biochem Behav 2004 77 567 573 10.1016/j.pbb.2003.12.019 15006468

[B86] García-Gutiérrez MS Manzanares J Overexpression of CB2 cannabinoid receptors decreased vulnerability to anxiety and impaired anxiolytic action of alprazolam in mice J Psychopharmacol 2011 25 111 120 10.1177/0269881110379507 20837564

[B87] García-Gutiérrez MS García-Bueno B Zoppi S Leza JC Manzanares J Chronic blockade of cannabinoid CB2 receptors induces anxiolytic-like actions associated with alterations in GABA(A) receptors Br J Pharmacol 2012 165 951 964 10.1111/j.1476-5381.2011.01625.x 21838753 PMC3312491

[B88] Pertwee RG GPR55: a new member of the cannabinoid receptor clan? Br J Pharmacol 2007 152 984 986 17876300 10.1038/sj.bjp.0707464 PMC2095104

[B89] Ryberg E Larsson N Sjögren S Hjorth S Hermansson NO Leonova J Elebring T Nilsson K Drmota T Greasley PJ The orphan receptor GPR55 is a novel cannabinoid receptor Br J Pharmacol 2007 152 1092 1101 17876302 10.1038/sj.bjp.0707460 PMC2095107

[B90] Kapur A Zhao P Sharir H Bai Y Caron MG Barak LS Abood ME Atypical responsiveness of the orphan receptor GPR55 to cannabinoid ligands J Biol Chem 2009 284 29817 29827 10.1074/jbc.M109.050187 19723626 PMC2785612

[B91] Sharir H Console-Bram L Mundy C Popoff SN Kapur A Abood ME The endocannabinoids anandamide and virodhamine modulate the activity of the candidate cannabinoid receptor GPR55 J Neuroimmune Pharmacol 2012 7 856 865 10.1007/s11481-012-9351-6 22454039 PMC3669693

[B92] de Fonseca FR Schneider M The endogenous cannabinoid system and drug addiction: 20 years after the discovery of the CB1 receptor Addict Biol 2008 13 143 146 10.1111/j.1369-1600.2008.00116.x 18482429

